# Editorial for the Special Issue on Analysis, Design and Fabrication of Micromixers II

**DOI:** 10.3390/mi13122176

**Published:** 2022-12-08

**Authors:** Kwang-Yong Kim

**Affiliations:** Department of Mechanical Engineering, Inha University, Incheon 22212, Republic of Korea; kykim@inha.ac.kr; Tel.: +82-010-3641-3096

Micromixers are important components of lab-on-a-chip systems, and also have many biological and chemical applications [1]. Micromixers are generally classified into two categories, i.e., active and passive micromixers, depending on the mechanism of mixing enhancement. Our previous Special Issue (Special Issue on Analysis, Design and Fabrication of Micromixers I) [2] reported mostly on passive micromixers, in addition to two electrokinetic micromixers. However, except for two review papers [3,4], the current Special Issue places a greater emphasis on active mixing, reporting on five active [5,6,7,8,9] and six passive [10,11,12,13,14,15] micromixers. Three of the active micromixers have an electrokinetic driving force [5,6,7], but the other two are activated by mechanical mechanisms [8] and acoustic streaming [9]. Three studies [5,13,14] employ non-Newtonian working fluids, one of which deals with nano-non-Newtonian fluids [13]. Most of the cases investigate micromixer design, except for ref. [5]. Additionally, most of the passive micromixers have three-dimensional (3D) obstructions [10,12,14] or 3D channel shapes [11,13]. Seven studies [5,6,9,12,13,14,15] are numerical, two [7,10] are experimental, and the other two are experimental with numerical validation [8] and numerical with experimental validation [11].

Hejazian et al. [3] reviewed previous studies on microfluidic mix-and-jet devices, which are efficient for sample delivery in biomolecular applications. They introduced designs, characteristics, and fabrication techniques. Experimental techniques for the analyses of mixing and jetting are also summarized. They expect innovative designs of these devices to appear in applications in many other fields, such as basic studies on physics and chemistry, polymer fabrication, and the kinetics of nanoparticles. The review paper by Sahadevan et al. [4], which was selected as a feature paper and an Editor’s Choice paper by this journal, reports on recent progress and future perspectives on microfluidic applications of artificial cilia, which have good capabilities in mixing, pumping, and particle handling. Microfluidics using artificial cilia can be an efficient tool to control fluid flow with high precision in lab-on-a-chip and other biomedical applications. They suggest that effective and economic manufacturing techniques must be developed to make artificial cilia more practical in these applications.

Banos et al. [5] examined the effects of combinations of non-Newtonian fluids, slippage, and finite-sized ions on the mixing of fluids in an oscillatory electroosmotic flow. They proposed a numerical model for the analysis of the mixing. The results revealed that the effects enhanced the mixing by about 90%. Shi et al. [6] proposed a novel electrokinetic micromixer with staggered electrodes based on light-actuated AC electroosmosis, and analyzed the mixing of fluids numerically using the finite element method. They performed a parametric study using parameters such as the width, length, and spacing of the electrodes, as well as the channel height to identify effects on mixing performance, and thus optimal micromixer geometry. Yang et al. [7] also proposed a new electrokinetic micromixer with a quasi T-channel and electrically conductive sidewalls. An experiment was carried out to find the effects of Reynolds number, AC voltage and frequency, and electric conductivity ratio on mixing performance. They suggested that the proposed micromixer showed higher mixing performance compared to the conventional micromixers with the electrodes located at the channel outlet.

Koike and Takayama [8] introduced a novel active micromixer design for the concentration control of the fluids. They developed a mechanical mechanism to produce concentration gradients in multiple main chambers, arranged parallel to the body channel. Each main chamber is surrounded by a driving chamber, and agitation is caused in the main chamber due to the expansion or contraction of the driving chamber. The fluid moving back and forth in the channel connecting the main chamber to the body channel controls the concentration. The concentration gradient was determined experimentally, but numerical simulation was also performed to confirm the mechanism of the micromixer. Although they could produce a concentration gradient, the concentration control was not so successful. An active micromixer using acoustic streaming was proposed by Endaylalu and Tien [9]. The acoustic streaming in a Y-junction microchannel was generated by introducing triangular structures at the channel walls. The mixing performance was evaluated numerically. A parametric study of the mixing performance was performed using the inlet velocity, triangular structure’s vertex angle, and oscillation amplitude. The results suggested that installing the triangular structure at the junction enhanced mixing by causing acoustic streaming. Additionally, lower inlet velocity, higher oscillation amplitude, and smaller vertex angle also improved the mixing performance.

Oevreeide et al. [10] proposed a novel passive micromixer design with double-curved structures located on a surface of the microchannel. In the experiment, confocal imaging was used to find the flow patterns. Additionally, analyzing the fluorescence pattern monitored the development of homogenization. They suggested that introducing a second curved structure to the simple curved structure had a remarkable effect on mixing performance. Rouhi et al. [11] investigated a passive micromixer with a non-planar spiral microchannel with various cross-sections. The mixing performance was evaluated numerically in a Reynolds number (Re) range of 0.001–50. An experiment was also performed to validate the numerical solution. In the numerical test, a large-angle outward trapezoidal cross-section showed the best mixing performance of up to 95%. Juraeva and Kang [12] presented a passive micromixer design with eight mixing units. Each mixing unit was composed of four alternatively arranged baffles. The mixing units were stacked in a cross-flow direction, and four different arrangements were tested: one-, two-, four-, and eight-step stacking. The mixing was analyzed numerically in a range of Re from 0.5 to 50. The numerical results suggested that the highest mixing was obtained with the eight-step stacking for Re > 2, but with the two-step stacking for Re < 2.

Tayeb et al. [13] numerically analyzed the characteristics of nano-non-Newtonian fluids in a novel passive micromixer with a 3D channel structure. Mixing and heat transfer between two heated fluids were investigated. How the concentration of Al_2_O_3_ nanoparticles affects the pressure drop and thermal mixing for a Re range from 0.1 to 25 was tested. The results revealed that higher nanofluid concentration and stronger chaotic advection improved the hydrodynamic and thermal performances over the whole Re range remarkably. Mahammedi et al. [14] also numerically examined the mixing of non-Newtonian fluids in Kenics micromixers for Re = 0.1–500. The Y-shaped micromixer consisted of six helical elements arranged alternately. They suggested that for the carboxymethyl cellulose solutions with power–law indices of 0.49–1, the Kenics mixer showed high mixing for both low and high Reynolds numbers. Juraeva and Kang [15] reported on the mixing performance of a modified Tesla micromixer. The design was modified by introducing a tip clearance above the wedge-shaped divider. The mixing performance was analyzed numerically for Re = 0.1–80. In order to measure the mixing performance, the degree of mixing at the micromixer outlet and the pressure drop through the mixer were used. They suggested that the tip clearance introduced in the modified design enhanced the mixing performance in a wide Re range.

I appreciate all the authors who contributed to this Special Issue. Additionally, thanks also go to the reviewers for the submitted papers and the editorial staff who conducted the review process. Further contribution to this topic can be made to the Topical Collection “Micromixers: Analysis, Design and Fabrication” of this journal.
